# Metastatic Malignant Peripheral Nerve Sheath Tumor (MPNST) in Neurofibromatosis Type 1: Challenges in Diagnosis and Management

**DOI:** 10.7759/cureus.26140

**Published:** 2022-06-21

**Authors:** Steven Pulliam, Kiran Madwani, Ashley D Fox, Nadia El-Hachoum, Asad Ullah, Nikhil Patel, Nagla A Karim

**Affiliations:** 1 Internal Medicine, Medical College of Georgia, Augusta University, Augusta, USA; 2 Pathology, Medical College of Georgia, Augusta University, Augusta, USA; 3 Medicine, Augusta University, Augusta, USA; 4 Hematology/Oncology, Georgia Cancer Center, Augusta University, Augusta, USA

**Keywords:** cancer-genetics, metastatic mpnst, neurofibromatosis type i, neurofibromatosis, malignant peripheral nerve sheath tumors

## Abstract

Malignant peripheral nerve sheath tumors (MPNSTs) are growths that arise in conjunction with a peripheral nerve and are believed to originate from neural crest cells. These tumors can arise sporadically but are often associated with the cancer-predisposing genetic condition, neurofibromatosis type 1 (NF-1). The clinical presentation of an enlarging mass, pain, paresthesias, and neurologic deficits can mirror that of other soft tissue sarcomas. Thus, clinical suspicion should remain high for an MPNST when this aggregation of symptoms arises, particularly in those with a genetic proclivity.

We report the case of metastatic MPNST in a 44-year-old female with a long history of NF-1. She was first seen for evaluation of progressive forearm and hand weakness associated with numbness and paresthesias in her second through fourth digits which prompted a need for an MRI. A forearm mass was discovered, and she underwent surgical intervention which revealed an MPSNT with positive margins. The patient completed radiation therapy for this lesion, but ultimately her forearm lesion recurred and progressed with metastasis to the lungs. Local recurrence was managed with a trans-humeral amputation and her systemic involvement necessitated chemotherapy. She was ultimately enrolled in a clinical trial for adult patients with recurrent advanced solid tumors.

Given the potentially fatal course of NF-1-associated MPNSTs, clinical suspicion should remain high and early diagnosis and intervention with regular clinical surveillance are of utmost importance in this patient population.

## Introduction

Neurofibromatosis is one of the most prevalent genetic conditions in today's society, estimated to affect one in every 3,500 people worldwide [1]. Cutaneous findings are the hallmark of this disease and include cafe-au-lait macules, skinfold freckling, and cutaneous neurofibromas. Additional diagnostic criteria include the presence of optic gliomas, iris hamartomas, and the diagnosis of NF-1 in a first-degree relative [1]. The condition is inherited in an autosomal dominant manner and arises due to a mutation in the neurofibromin 1 gene on chromosome 17. The protein product of this gene, neurofibromin, is ubiquitously expressed and heavily involved in regulating the Ras proto-oncogene, a driver of cell growth and differentiation found to be mutated in a variety of cancers [2]. Given the lack of suppression of this growth-promoting signal pathway, it is no surprise that patients diagnosed with NF-1 are greatly more likely to develop malignancies, with an estimated lifetime cancer risk of 59.6% [3].

One such neoplasm affecting these patients is the malignant peripheral nerve sheath tumor (MPNST). These soft tissue sarcoma variants occur at a rate of 1:3,500 in those with NF-1, a greatly elevated risk compared to the 1:100,000 rate seen in the general population. It has been estimated that anywhere from 8% to 13% of NF-1 patients will develop these tumors during their lifetime, and, more importantly, this malignancy has been reported as the leading cause of mortality in this patient population [3].

These tumors account for 10% of all malignant sarcomas and half of all MPNSTs occur in patients diagnosed with NF-1. The clinical presentation is similar to that of other soft tissue sarcomas, presenting as an enlarging mass over a period of months. The most common origin of these tumors is from nerve roots and bundles in the extremities, particularly the brachial and sacral plexus. Thus, the vast majority of MPNSTs arise in the proximal aspects of the upper and lower extremities with associated neurologic deficits, pain, and paresthesias. Clinical suspicion should remain high for an MPSNT when this conglomeration of symptoms is present. This is particularly true in those diagnosed with NF-1, as new-onset pain in a pre-existing neurofibroma is highly indicative of an MPSNT and should prompt immediate investigation [4].

The current standard of care varies depending on the location and degree of systemic involvement. Localized, primary tumors are managed with surgical resection and concomitant radiation, while metastatic lesions often undergo chemotherapy and, if possible, resection [5]. Regardless of which intervention is pursued outcomes remain poor. Local recurrence occurs in 40-65% of surgically excised cases and overall rates of metastasis transpire similarly at 30-60%. In fact, five-year survival rates have been reported to range from 20% to 50%, with a mortality rate approaching 75% [5].

Given the potentially fatal course of NF-1-associated malignancies, early diagnosis and regular clinical surveillance are of utmost importance. Herein, we introduced the case of a 44-year-old female who was diagnosed with NF-1 at a young age and subsequently developed an MPNST with metastatic lesions. We discussed her clinical course and associated radiology, pathology, and surgical findings.

## Case presentation

A 43-year-old female with a past medical history of neurofibromatosis type 1 (NF-1) presented to the plastic surgery clinic with complaints of weakness in her right hand and forearm associated with numbness and tingling in her second, third, and fourth digits that had progressively worsened over two years. On physical examination, a large, firm mass was noted in the right forearm with associated numbness and paresthesias in the fingers extending from the second digit to the radial aspect of the fourth digit. Laboratory evaluation at this visit revealed no abnormal findings. A non-contrast MRI was ordered to evaluate for median nerve involvement which revealed a large, heterogenous oval-shaped tumor in the proximal forearm extending from the level of the elbow joint distally. The median nerve could be visualized in the carpal tunnel and proximally for 5 cm before the soft tissue distortion associated with the tumor obscured further tracing of the nerve.

Surgical removal was planned with possible nerve reconstruction due to the symptoms of compressive neuropathy and the sizeable mass. The forearm mass was dissected using a volar approach and it was discovered that the mass was originating from the median nerve. Once the mass had been completely excised, the nerve was repaired with neural tubing under microscopy and the case proceeded without any further events. The final diagnosis of this lesion revealed a malignant peripheral nerve sheath tumor (MPNST), and the patient was referred to an oncologist for radiation therapy which was completed soon after surgery.

**Figure 1:**
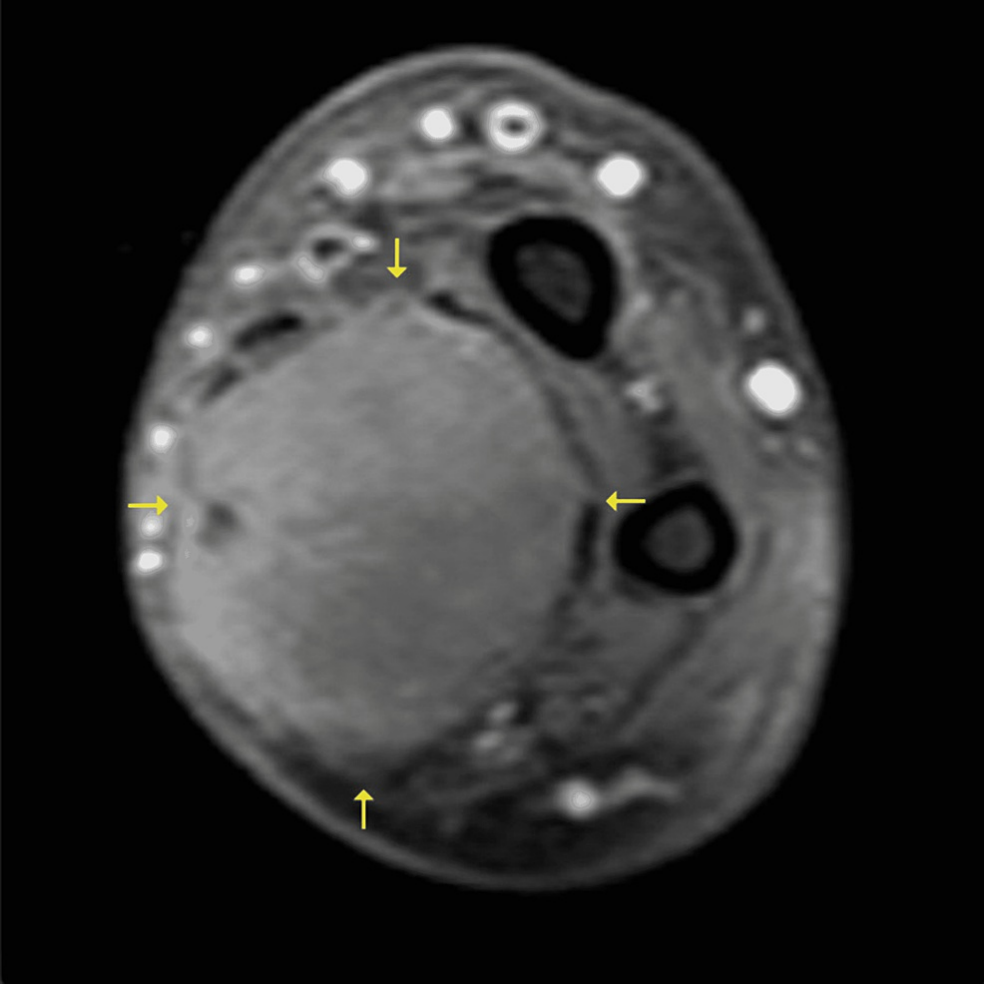
MRI showing a large, heterogeneously enhancing mass (arrows) in the right forearm with small, internal foci indicative of necrosis.

Despite this adjuvant therapy, the forearm mass recurred and progressed over the course of the following two years and the patient developed metastatic lesions in the right buttock and vulva requiring excision and chemotherapy. At that time, a follow-up MRI of the forearm revealed a marked increase in size of an enhancing heterogeneous mass in the proximal aspect of the resection bed consistent with progression. The mass measured 11.6 x 8.3 x 7.5 cm compared to 4.7 x 4.0 x 3.2 cm previously (Figure 1). The patient’s case was then discussed at a multidisciplinary tumor board and the decision was made to pursue a trans-humeral amputation to prevent further progression of the localized forearm mass. The case proceeded without any complications and subsequent gross and histopathologic examinations were performed.

**Figure 2:**
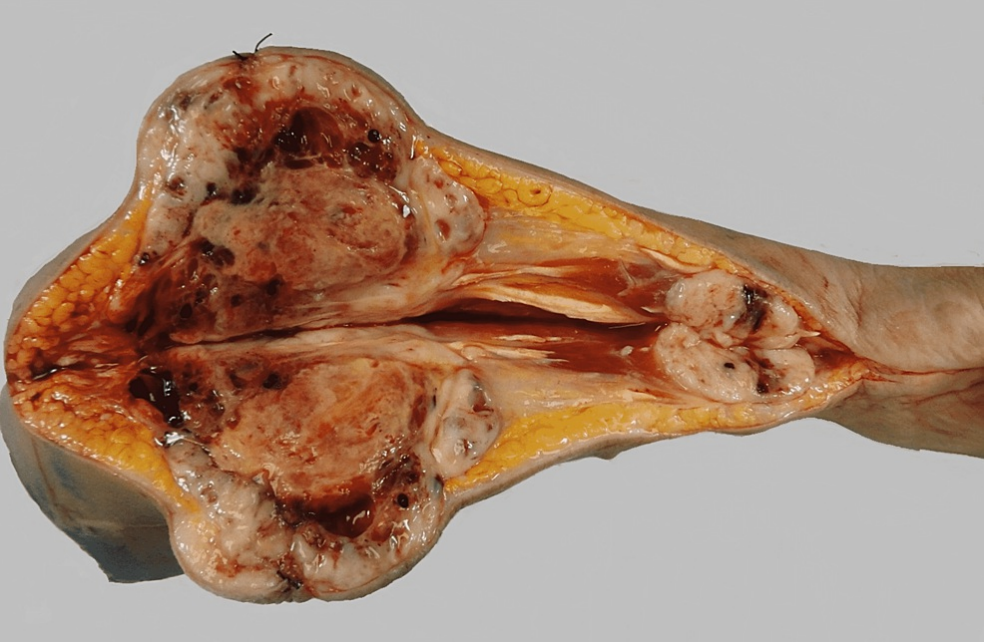
The gross image of the right lower arm with two tumors showing well-circumscribed tan, white nodules with hemorrhagic and focal necrotic areas.

Grossly, the specimen showed two distinct subcutaneous well-circumscribed tan, white lesions with focal areas of hemorrhage and necrosis extending into deep soft tissue and muscle layer. The distal (closest to the wrist) nodule was measured at 5.0 cm and was intermingled with muscle bundles. The larger proximal (closest to the resection margin) nodule was measured at 12.0 cm and was also intermingled with muscle bundles (Figure 2).

**Figure 3:**
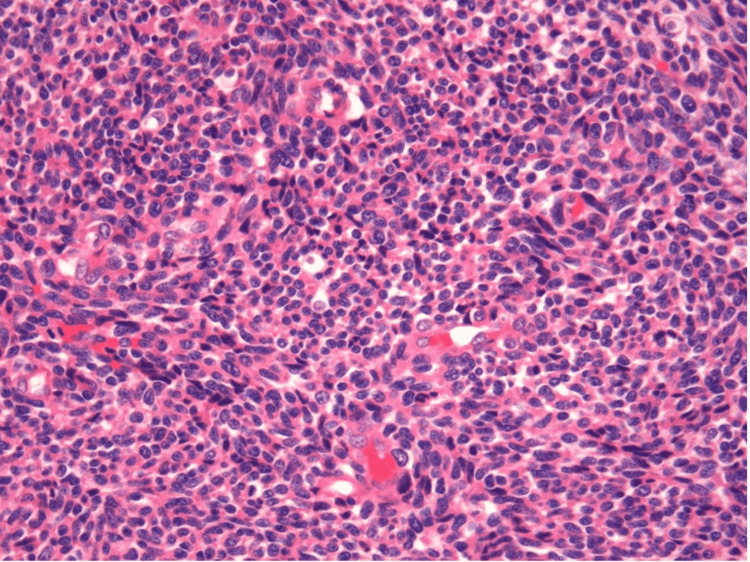
Haphazardly arranged fascicles of the spindle to epithelioid cells with mild nuclear hyperchromasia, pleomorphism, and eosinophilic cytoplasm (H and E, 20x).

Histopathological examination revealed spindle to epithelioid cells and haphazardly arranged fascicles with mild cytologic atypia and nuclear hyperchromasia. The cells revealed abundant eosinophilic cytoplasm with identifiable mitosis (Figure 3). Based on the patient’s previous history and histologic examination the final diagnosis of pT2 staged high-grade malignant peripheral nerve sheath tumors was rendered.

**Figure 4:**
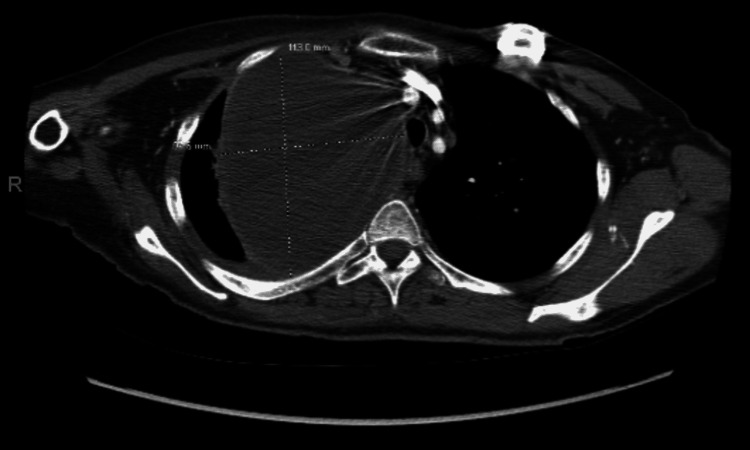
Chest CT showing a large, moderately enhancing mass in the right upper lobe.

On surveillance imaging with positron emission tomography (PET)/CT approximately seven months later, the patient was found to have developed a new lung mass. Imaging revealed a moderately enhancing right upper lobe pulmonary mass measuring 8.2 x 9 x 10.7 cm that directly invaded the right mediastinum as well as the anterior and posterior right pleura (Figure 4).

**Figure 5:**
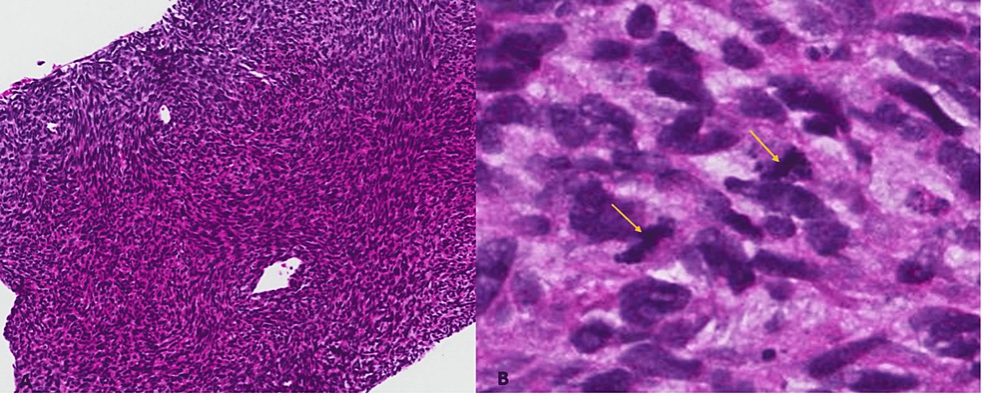
Cellular spindle cell neoplasm in haphazardly arranged fascicles (H and E, 10x) (A). Spindle to epithelioid cells with nuclear hyperchromasia and pleomorphism with identifiable mitosis (arrow) (H and E, 40x) (B).

**Figure 6:**
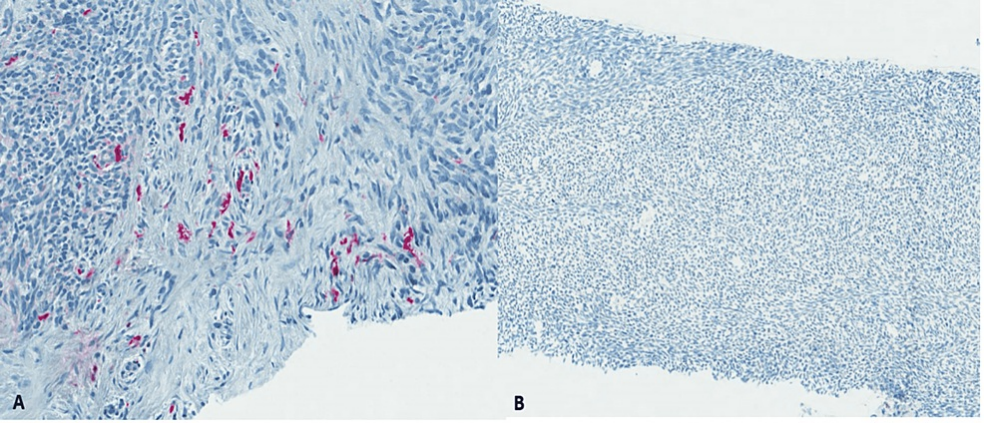
Patchy staining of tumor cells (S100, 20x) (A). Tumor cells are negative for pankeratin (S100, 10x) (B).

This mass was biopsied and revealed a spindle cell lesion with nuclear atypia and hyperchromasia and identifiable mitosis. The tumor was found to be patchy positive for S100 and negative for pankeratin, supporting the diagnosis of MPNST (Figures 5, 6).

The lung mass was deemed unresectable, and the patient’s case was again discussed at a multidisciplinary tumor board. Her metastasis was rapidly progressive with additional metastasis to bowel, pancreas, left adrenal gland, liver, and bone. Ultimately, the patient developed severe cachexia and significant cancer-related pain which was difficult to manage. She underwent multiple hospital admissions for numerous reasons including sepsis due to urinary tract infections, dehydration, and acute kidney injury, and lastly, malignant gastric outlet obstruction and jejunojejunal intussusception for which patient did not want to undergo any invasive procedures, including palliative stenting by gastroenterology or percutaneous endoscopic gastrostomy (PEG)-tube placement by surgery. At this time, the patient elected to pursue hospice care given the terminal nature of her condition.

## Discussion

Neurofibromatosis type 1 is associated with several types of malignant tumors, with a recent study showing that persons with NF-1 being nearly 10 times more likely to develop any type of cancer during their lifetime compared to the general population [6]. The cancer types identified to occur more commonly in NF-1 patients included gliomas, MPNST and other sarcomas, breast cancers, ovarian cancer, melanoma, prostate cancer, and pheochromocytoma and other neuroendocrine tumors. Of these, it was demonstrated that gliomas, MPNSTs, and breast cancer developed at a younger age in patients with NF-1. Additionally, it was found that undifferentiated pleomorphic sarcoma, high-grade glioma, MPNSTs, ovarian cancer, and melanoma were found to be more deadly to persons with NF-1. In this particular study, MPNSTs developed in ∼15% of the patients with NF-1.

MPNST is a malignant tumor that can affect people of all ages, but most commonly occurs in younger patients diagnosed with NF-1. The most common presentation includes a combination of pain, paresthesias, neurologic deficits, and an enlarging mass [7]. Several factors have been associated with their development including a microdeletion of the NF-1 gene, radiation exposure, presence of subcutaneous neurofibromas, and a personal/family history of MPNST [8].

Diagnosis of MPNSTs has remained challenging, given the mirrored presentation of this tumor to both benign neurofibromas and other malignant soft tissue sarcomas. Imaging studies such as CT and MRI have routinely been implemented to define tumor size and degree of invasion, while PET scans have shown higher efficacy in distinguishing between benign and malignant masses [9]. Despite the utility of these studies, histopathologic examination remains the diagnostic gold standard. The proliferation of spindle cells in a fascicular growth pattern with alternating degrees of cellularity is characteristic of MPNST histologically [10].

Once diagnosed, current recommendations focus on complete excision of the neoplasm with wide negative margins, as this is a strong predictor of survival. Those with negative margins were found to have respective three-year and five-year survival rates that were 27% and 45% higher compared to those with positive margins. The anatomic region of the tumor was also noted to significantly impact the frequency of positive margins, with the highest rates occurring in the pelvis (42%) and chest (32%) [5]. In addition to surgical resection, significant clinical improvement has been reported with adjuvant radiation therapy. Kahn et al. revealed a 15.7-month improvement in median survival in NF-1-associated cases of MPNST that underwent radiation therapy [11].

Despite the success achieved in managing these tumors with resection and radiation, no standardized systemic therapies currently exist. This represents a prime target for genomic-centered therapy. As previously discussed, patients with NF-1 have an inactivating modification in their neurofibromin protein. This alteration removes the inhibitory effect of neurofibromin on the rat sarcoma virus/rapidly accelerated fibrosarcoma (RAS/RAF) signaling cascade, ultimately allowing for unfettered growth. Recent studies have revealed additional genes and signaling pathways implicated in cases of both wild-type and NF-1-associated MPNSTs, including cyclin-dependent kinase inhibitor 2A (CDKN2A), proto-oncogene B-Raf (BRAF), and phosphoinositide 3-kinase/phosphatase and tensin Homolog/protein kinase B (PI3K/PTEN/AKT).

In a cohort of 186 MPNSTs, Kaplan et al. reported the most commonly implicated mutations were found in DNA repair genes (70%), CDKN2A (57%), and the PI3 pathway (46%). When subdivided into NF-1-associated and wild-type tumors, CDKN2A mutations were found to occur more frequently in NF-1 cases (71% vs 34%, respectively) and those with NF-1 alterations were also found to have 2.5 times as many mutations in their PI3 pathway. Alterations in the BRAF gene were also reported at a rate of 10% and were almost exclusively found in wild-type tumors [12].

The involvement of each of these genes and pathways represents a much-needed therapeutic target for those diagnosed with MPNSTs. The previously established involvement of the RAS/RAF pathway in these tumors highlights the importance of targeting this pathway in particular. Response to inhibitors of the downstream targets BRAF and mitogen-activated protein kinase (MEK) have been reported by Kaplan et al. in cases of MPNSTs exhibiting BRAF mutations [12]. Although current clinical data regarding the utility of these therapies in cases of MPNSTs is limited, clinical trials evaluating this targeted therapy are currently undergoing. Dombi et al. also reported the efficacy of MEK inhibitors in treating NF-1-associated benign plexiform neurofibromas [13]. Given the response of this malignancy to MEK-targeted therapy, further investigations should be pursued to evaluate the utility of this therapy in managing additional NF-mutant neoplasms, such as MPNSTs.

## Conclusions

The present study has reported an interesting phenomenon, metastatic MPNST in the setting of NF-1. Given the predisposition of those with NF-1 for malignancies, these patients should undergo regular medical surveillance and it is crucial to be aware of the presenting symptoms of MPNSTs. Interventions should occur early and aggressively, as wide, negative margins in conjunction with radiation therapy have shown significant improvement in patient survival. Given the complex nature of these tumors, multidisciplinary management should also be implemented and include surgical, radiation, and medical oncologists, as well as pathologists well-versed in sarcomas.

Genomic profiling should also be pursued, as several genes have been isolated as potential therapeutic targets including RAS, BRAF, and MEK. Additionally, further investigations should be pursued in evaluating therapies that can target the frequently reported alterations in the CDKN2A pathway and DNA repair genes in cases of MPNSTs These discoveries have opened the door to providing a personalized approach to managing these aggressive tumors.
